# Supplementary material for: The Bioactive Gamma-Oryzanol from Oryza sativa L. Promotes Neuronal Differentiation in Different In Vitro and In Vivo Models
Source: Antioxidants (Basel). 2024 Aug 9;13(8):969. doi: 10.3390/antiox13080969 (PMC11352202; doi:10.3390/antiox13080969)
Supplement: Supplementary file 1 [file antioxidants-13-00969-s001.zip › antioxidants-3026010-supplementary.pdf]

Supplementary Material

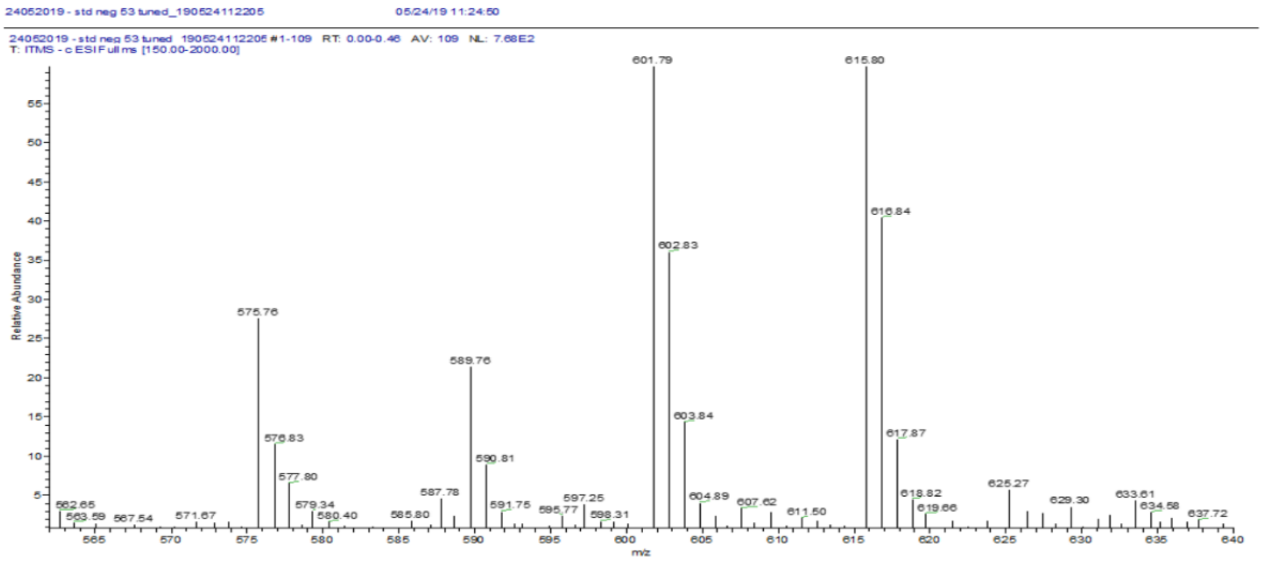

Supplementary Figure S1: Spectrum of all gamma-oryzanol components analyzed with mass-spectrometry.

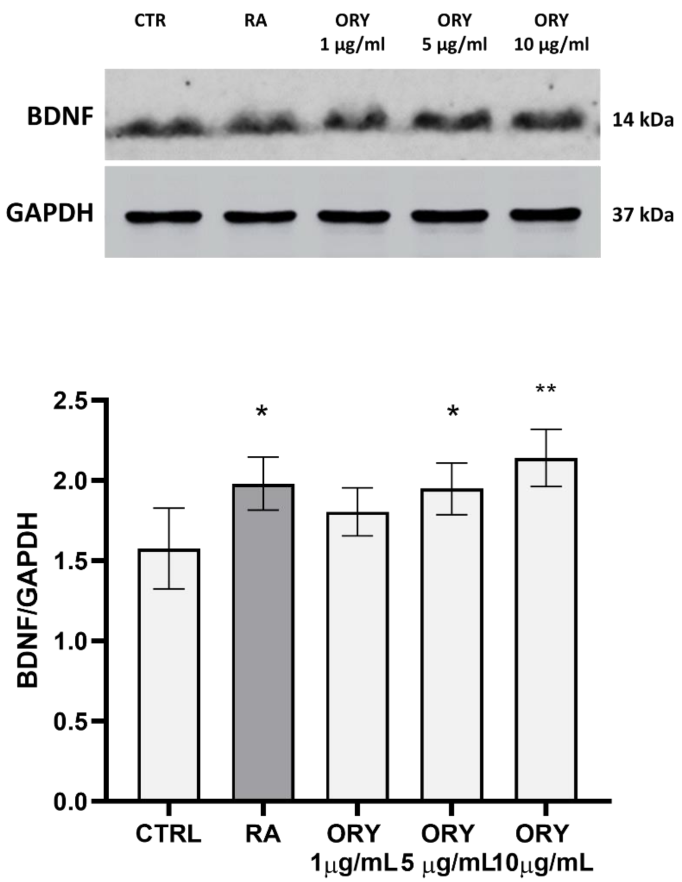

Supplementary Figure S2: Representative western-blot analysis of mature BDNF protein expression in SH-SY5Y treated with vehicle, RA or ORY.

SH-SY5Y have been treated with vehicle or RA or ORY at 1, 5 or 10 µg/ml for 5 days. Data are represented as mean ± S.E.M. and normalized over GAPDH protein expression. Statistical significance was assessed by ONE Way ANOVA. ** $p < 0.01$; * $p < 0.05$.

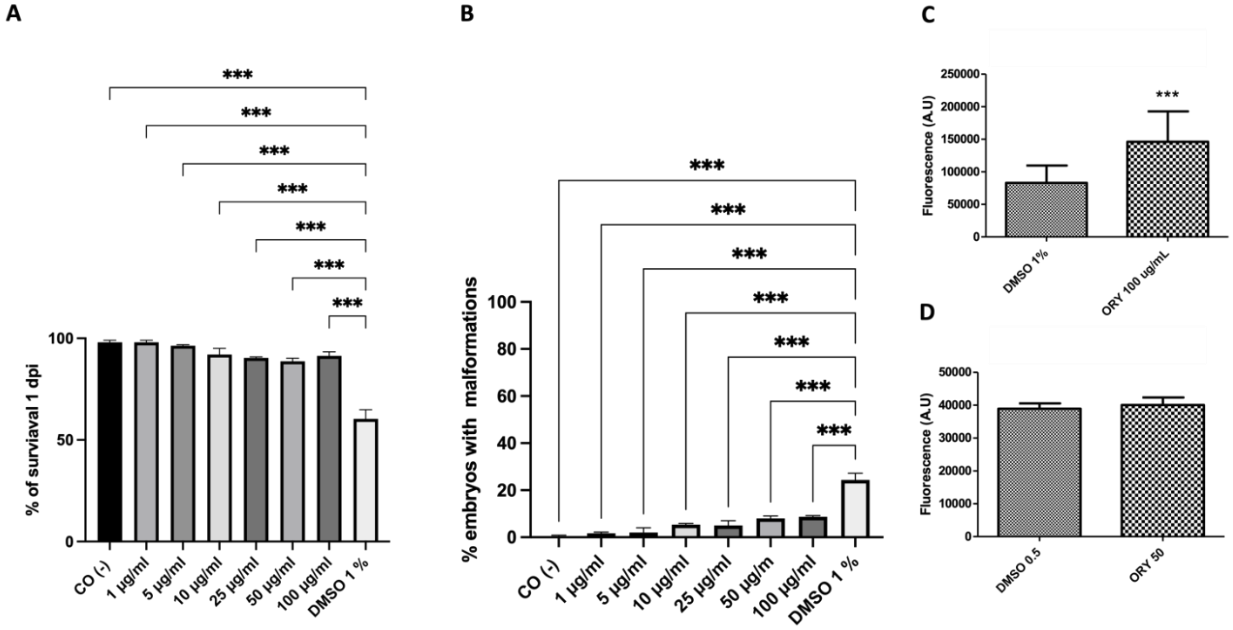

Supplementary Figure S3: Dose setting for ORY injection.

A) Survival at one-day post injection (dpt). B) Evaluation of morphological abnormalities post ORY injection. C-D) Fluorescence intensity in 48 hpf embryos injected with DMSO and ORY at C) 100µg/ml or D) 50 µg/ml. Data are presented as Mean+/- SD. Statistical significance was assessed by ONE Way ANOVA (A-B) or Unpaired T test (C-D). CO (-) indicates the negative control of the experiments represented by uninjected embryos. *** $p < 0.001$.

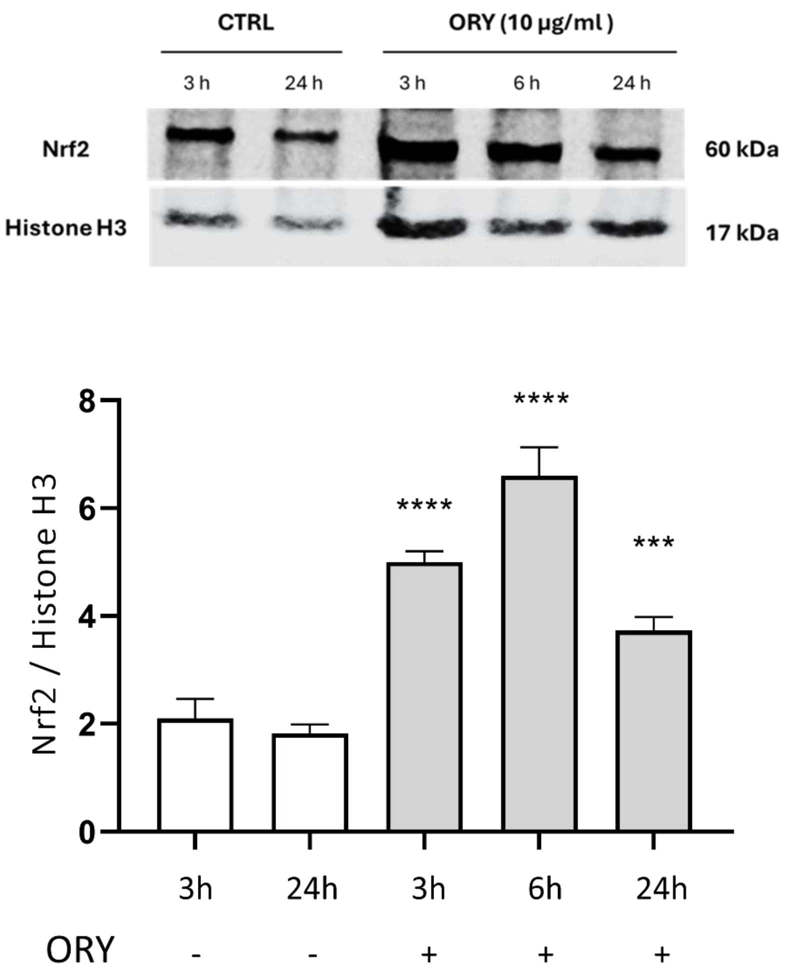

Supplementary Figure S4: Nrf2 nuclear translocation induced by ORY in SH-SY5Y.

SH-SY5Y cells were treated or untreated with ORY at 10 µg/ml 3, 6, and 24 h. Nuclear fractions were isolated as described in the Material and Methods. Nuclear expression of Nrf2 was assessed by Western blotting and Histone H3 expression was used as loading control. Data are represented as mean ± SD; ****$p \leq 0.0001$ and ***$p \leq 0.001$, versus untreated cells
